# The relationship between spino-pelvic alignment and primary dysmenorrhea

**DOI:** 10.3389/fsurg.2023.1125520

**Published:** 2023-02-08

**Authors:** Juehan Wang, Xin He, Ce Zhu, Hong Ding, Ganjun Feng, Xi Yang, Limin Liu, Yueming Song

**Affiliations:** ^1^ Department of Orthopedic Surgery and Orthopedic Research Institute, West China Hospital, Sichuan University, Chengdu, China; ^2^ Department of Obstetrics and Gynecology, Ruijin Hospital, Shanghai Jiaotong University School of Medicine, Shanghai, China

**Keywords:** primary dysmenorrhea, sagittal spino-pelvic alignment, global sagittal balance, pathological mechanisms, sacral slope

## Abstract

**Introduction:**

Most women of reproductive age suffered from the primary dysmenorrhea (PD). Up to date, most studies on the etiology of dysmenorrhea focused on endocrine factors while ignored the effect of spino-pelvic bony anatomy on uterus. In this study, we innovatively shed light on the relationship between primary dysmenorrhea and sagittal spino-pelvic alignment.

**Materials and Methods:**

120 patients diagnosed with primary dysmenorrhea and a control group of 118 healthy volunteers were enrolled into this study. All subjects received the standing full-length posteroanterior plain radiography to evaluate the sagittal spino-pelvic parameters. The visual analog scale (VAS) was used to assess pain rating of primary dysmenorrhea patients. Analysis of variance (ANOVA) or Student's t test was performed to measure statistical significance between differences.

**Results:**

There was a significant difference in pelvic incidence (PI), sacral slope (SS), lumbar lordosis (LL) and thoracic kyphosis (TK) between PD group and Normal group (*P*<0.05). Furthermore, in PD group, the PI and SS was significant different between mild pain group and moderate pain group (*P*<0.05) and there was a significant negative correlation between pain rating and SS. From the perspective of sagittal spinal alignment, the majority of PD patients were classified with Roussouly type 2, meanwhile most normal people were classified with Roussouly type 3.

**Conclusion:**

Sagittal spino-pelvic alignment was related to primary dysmenorrhea symptoms. Lower SS and PI angles may contribute to a worsen pain in PD patients.

## Introduction

Primary dysmenorrhea (PD) is one of the most complained diseases among adolescence and childbearing age female. Women of reproductive age are reported to suffer from dysmenorrhea to the extent of 16%-91%, with a higher incidence in adolescents observed in 56.1% to 93.0% (1–4). In addition to the high prevalence rate, a series of related symptoms often lead to impairment in quality of life (5–7). The symptoms associated with dysmenorrhea include gastrointestinal symptoms such as nausea, bloating, diarrhea, constipation, or both, along with vomiting and indigestion. Also, irritability, headache, and low back pain are prevalent among women presenting with primary dysmenorrhea.

The etiology of PD is not fully elucidated. Since no organic lesion was detected in primary dysmenorrhea, there were various possible pathogenic factors reported in the previous literature such as overproduction of uterine PGs, psychological factors, vasoconstriction caused by the increase of vasopressin level and adverse uterine position (8).

However, patho-physiologic rather than anatomical factors are more stressed in the present literature discussing the etiology of PD. Since Roussouly etal. reported the classification of the normal variation in the sagittal alignment of the human lumbar spine and pelvis (9), the spino-pelvic sagittal alignment has attracted more and more attention in recent years. Spinopelvic imbalance may lead to low back pain(LBP) and LBP-related disabilities (10, 11). There are no studies now have been reported on the relationship between primary dysmenorrhea and the sagittal lumbar-pelvic alignment. The mismatch lumbar-pelvic parameters may affect the muscleskeleton system of the pelvis cavity, which could further lead to the adverse uterine position, and may be one of the causes of dysmenorrhea in some women. This study was aimed to illustrate the relationship between lumbar-pelvic alignment and primary dysmenorrhea.

## Patients and methods

A retrospective study design was conducted to investigate the relationship between spino-pelvic alignment and primary dysmenorrhea.We enrolled 120 patients (24.7 ± 3.8 years old) diagnosed with primary dysmenorrhea and a control group of 118 healthy volunteers (23.5 ± 3.6 years old) without any uterine diseases or musculoskeletal disorders such as low back pain in the outpatient clinic of our department of West China hospital from July 2018 until May 2021. The exclusion criteria were: 1. Scoliosis or any congenital deformity of lumbosacral segment; 2. history of spinal or abdominal surgery; 3. smoking; 4. history of anxiety, depression, or severe mental disease; 5. obesity, defined as body mass index (BMI) > 25.0; and 6. history of taking oral contraceptive pills; 7. history of delivery.; 8. history of gynecological disease. All experimental procedures and methods were approved by the research ethics committee of the West China hospital and provided informed consent. All protocols were conducted in accordance with the research principles set forth in the Declaration of Helsinki.

### Clinical symptoms assessment

The visual analog scale (VAS) was used to assess lower abdominal pain of primary dysmenorrhea. And the patients were divided into three groups: (1) mild pain (1–3 scores), which can be tolerated and have little affect on daily life and sleep quality; (2) moderate pain (4–6 scores), evident pain affecting sleep but tolerable after simple treatment; (3) severe pain (7–10 scores), progressively intensive pain that is intolerable and requires specialized treatment for its relief.

### Radiographic assessment

**Figure 1:**
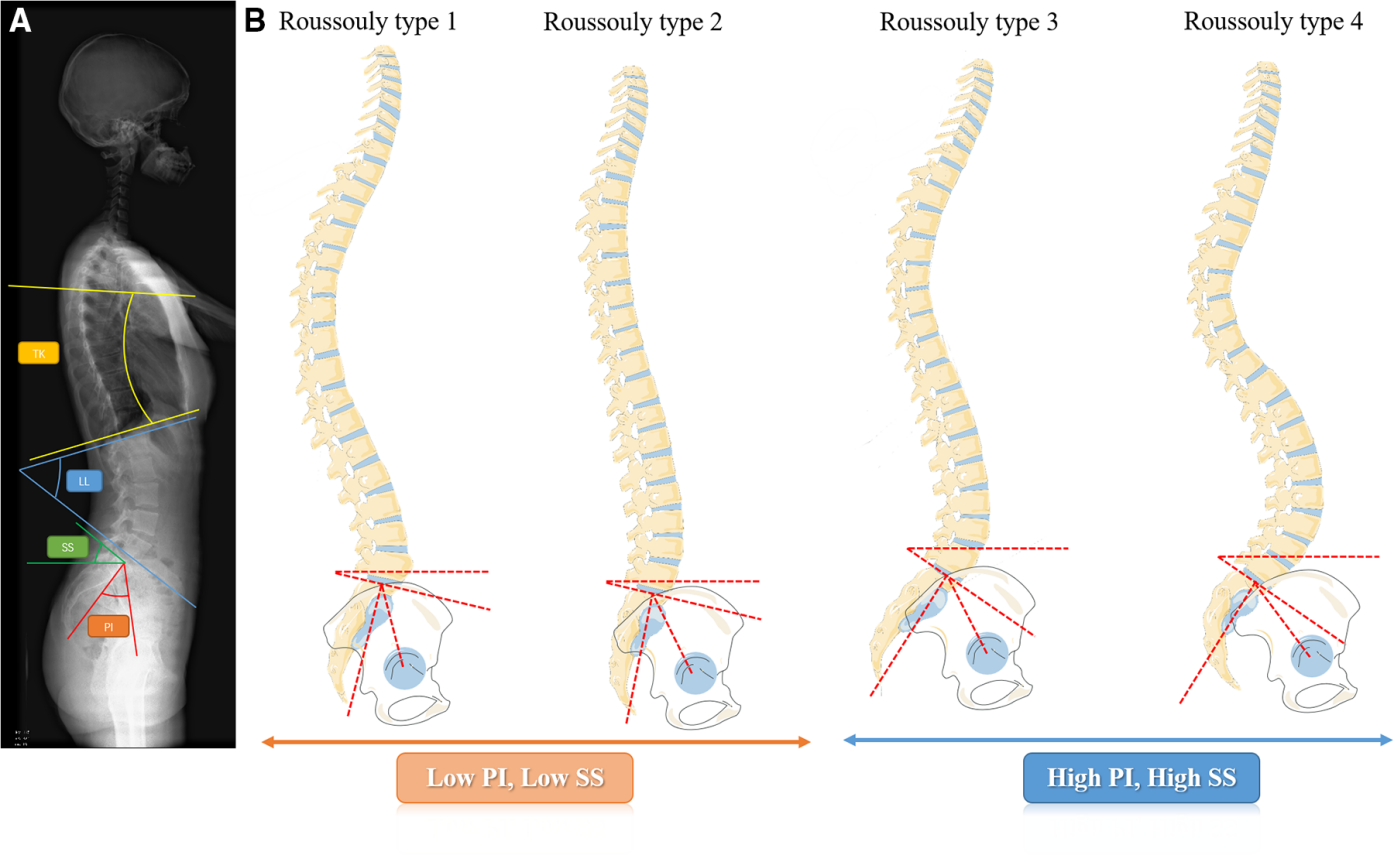
(**A**) The spino-pelvic parameters measured in the full-length plain radiography; (**B**) the roussouly classification: type 1: the sacral slope is less than 35°. The apex of the lumbar lordosis is located in the center of L5 vertebral body, The lower arc of lordosis is minimal, showing an incongruous long thoracolumbar kyphosis and short lumbar lordosis curve. Type 2: The sacral slope is less than 35°. The apex of the lumbar lordosis is located at base of the L4 vertebral body. The lower arc of lordosis is relatively flat. Type 3: The sacral slope is between 35° and 45°. The apex of lumbar lordosis is located at the center of the L4 vertebral body, showing a coordinated curve of almost equal length of thoracic kyphosis and lumbar lordosis. Type 4: the sacral slope is greater than 45°, which is associated with a high pelvic incidence. The apex of the lumbar lordosis is located at the base of the L3 vertebral body or higher, showing a coordinated longer lumbar lordosis and a shorter, larger thoracic kyphosis curve.

All subjects underwent standing full-length posteroanterior plain radiography. All radiological parameters were measured by two attending spinal surgeons, and the average values were adopted. The radiographic measurements included the following: lumbar lordosis (LL) was measured as the angle between the upper end plates of L1 and S1; Thoracic kyphosis (TK) was measured as the angle between the upper endplate of T5 and the lower endplate of T12; C7-sagittal vertical axis (SVA) was measured as the distance between the C7 plumb line and the posterosuperior border of S1; pelvic incidence (PI) was measured as the angle between a vertical line perpendicular to the sacral endplate at its midpoint and a line connecting the midpoint of the sacral endplate and the midpoint of a line connecting the centers of the two femoral heads; sacral slope (SS) was measured as the angle between the horizontal plane and the sacral plate (Figure 1A). And the PD patients were grouped by radiographic results according to the Roussouly classification (9) (Figure 1B).

### Statistical analysis

Statistical analyses were performed using GraphPad Prism (GraphPad Prism 8; GraphPad). The measurement data are expressed as mean ± standard deviation. Analysis of variance (ANOVA) or Student's *t* test was performed to measure statistical significance between differences, correlations were assessed using Pearsons correlative analysis, and *P* < 0.05 was considered statistically significant.

## Results

Table 1 shows the general characteristics of the primary dysmenorrhea group and the normal group. There were no statistically significant differences.

**Table 1:** Characteristics of the subjects (*N* = 238).

	PD group (*N* = 120)	Normal group (*N* = 118)
Age (years)	24.7 ± 3.8	23.5 ± 3.6
Height (cm)	162.63 ± 5.71	162.48 ± 7.29
Weight (kg)	55.29 ± 7.20	56.41 ± 6.57
BMI (Kg/m^2^)	20.96 ± 2.97	21.50 ± 3.17
Menstrual cycle length (days)	25.6 ± 3.0	25.4 ± 2.8
Duration of bleeding (days)	5.3 ± 1.1	5.1 ± 1.7

PD, primary dysmenorrhea.

### Comparison of spino-pelvic alignment between PD and normal groups

The parameters of lumbar-pelvic alignment between PD and normal groups were shown in Table 2. There was a significant difference in PI, SS, LL and TK between PD group and Normal group (*P*<0.05). The PI of PD group was 47.78 ± 5.60° vs. 52.38 ± 4.63° of Normal group (*P* = 0.000), the SS of PD group was 36.61 ± 7.57° vs. 39.61 ± 5.47° of Normal group (*P* = 0.0006), and the LL of PD group was 44.19 ± 7.07° vs. 48.44 ± 3.90° (*P* = 0.000), the TK of PD group was 20.08 ± 8.00° vs. 23.34 ± 7.19° of Normal group (*P* = 0.001). Whereas, there was no significant difference in SVA between PD group and Normal group the SVA of PD group was 11.35 ± 5.08 mm vs. 11.19 ± 5.41 mm of Normal group (*P* = 0.814).

**Table 2:** Results of sagittal spino-pelvic alignment parameters between PD and normal groups.

	PD group (*N* = 120)	Normal group (*N* = 118)	*P* value
Pelvic incidence (°)^a^	47.78 ± 5.60	52.38 ± 4.63	0.000
Sacral slope (°)^a^	36.61 ± 7.57	39.61 ± 5.47	0.000
Lumbar lordosis (°)^a^	44.19 ± 7.07	48.44 ± 3.90	0.000
Thoracic kyphosis (°)^a^	20.08 ± 8.00	23.34 ± 7.19	0.001
SVA (mm)	11.35 ± 5.08	11.19 ± 5.41	0.814

^a^ indicates statistically significant differences (*p* < 0.05).

### Analysis of the spino-pelvic alignment parameters in the PD subgroups divided according to the severity of pain

**Figure 2:**
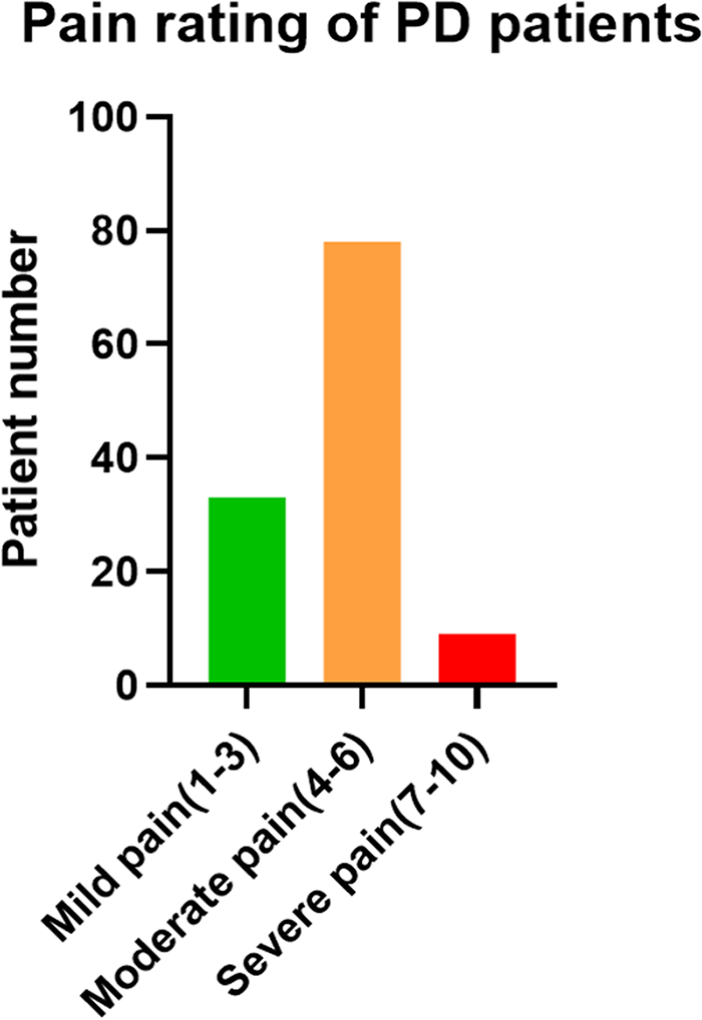
The PD patient number of each pain rating.

The PD patients were divided into three groups according to VAS scores. As shown in Figure 2, there were 33 patients with mild pain, 78 with moderate pain, and 9 with severe pain. Spino-pelvic parameters were analyzed among each group. The PI was significant different between mild pain group and moderate pain group (49.98 ± 6.66° vs. 47.28 ± 4.98°, *P* = 0.046), however, there was no significant difference between moderate pain group and severe pain group(47.28 ± 4.98° vs. 44.03 ± 3.57°, *P* = 0.208). The SS was also significant different between mild pain group and moderate pain group (42.64 ± 6.53°vs. 34.66 ± 6.58°, *P* = 0.000), whereas, no significant difference was found between moderate pain group and severe pain group (34.66 ± 6.58°vs. 33.06 ± 9.19°, *P* = 0.342). For LL,TK, and SVA, none of these differences were found statistically significant. The LL in each group were 45.51 ± 6.68°, 43.73 ± 6.93° and 43.30 ± 9.64° (45.51 ± 6.68° vs. 43.73 ± 6.93°, *P* = 0.281; 43.73 ± 6.93° vs. 43.30 ± 9.64°, *P* = 0.981). The TK in each group were 21.41 ± 4.38°, 19.34 ± 8.83° and 21.63 ± 10.55° respectively (21.41 ± 4.38° vs. 19.34 ± 8.83°, *P* = 0.431; 19.34 ± 8.83° vs. 21.63 ± 10.55°, *P* = 0.696). The SVA in each group were 10.90 ± 5.30 mm, 11.74 ± 4.92 mm and 9.68 ± 5.83 mm (10.90 ± 5.30 mm vs. 11.74 ± 4.92 mm, *P* = 0.710; 11.74 ± 4.92 mm vs. 9.68 ± 5.83 mm, *P* = 0.487). The results above were illustrated in Table 3.

**Table 3:** Results of sagittal spino-pelvic alignment parameters between mild to severe pain subgroups in PD patient.

	Mild pain group (*N* = 33)	Moderate pain group (*N* = 78)	Severe pain group (*N* = 9)	*P* value
Pelvic incidence (°)	49.98 ± 6.66^a^	47.28 ± 4.98^a^	44.03 ± 3.57	*P*_1 _= 0.046 *P*_2 _= 0.208
Sacral slope (°)	42.64 ± 6.53^a^	34.66 ± 6.58^a^	33.06 ± 9.19	*P*_1 _= 0.000 *P*_2 _= 0.342
Lumbar lordosis (°)	45.51 ± 6.68	43.73 ± 6.93	43.30 ± 9.64	*P*_1 _= 0.281 *P*_2 _= 0.981
Thoracic kyphosis (°)	21.41 ± 4.38	19.34 ± 8.83	21.63 ± 10.55	*P*_1 _= 0.431 *P*_2 _= 0.696
SVA (mm)	10.90 ± 5.30	11.74 ± 4.92	9.68 ± 5.83	*P*_1 _= 0.710 *P*_2 _= 0.487

^a^ indicates statistically significant differences (*p* < 0.05); *P*_1 _= Mild pain group *vs.* Moderate pain group, *P*_2 _= Moderate pain group *vs.* Severe pain group.

**Figure 3:**
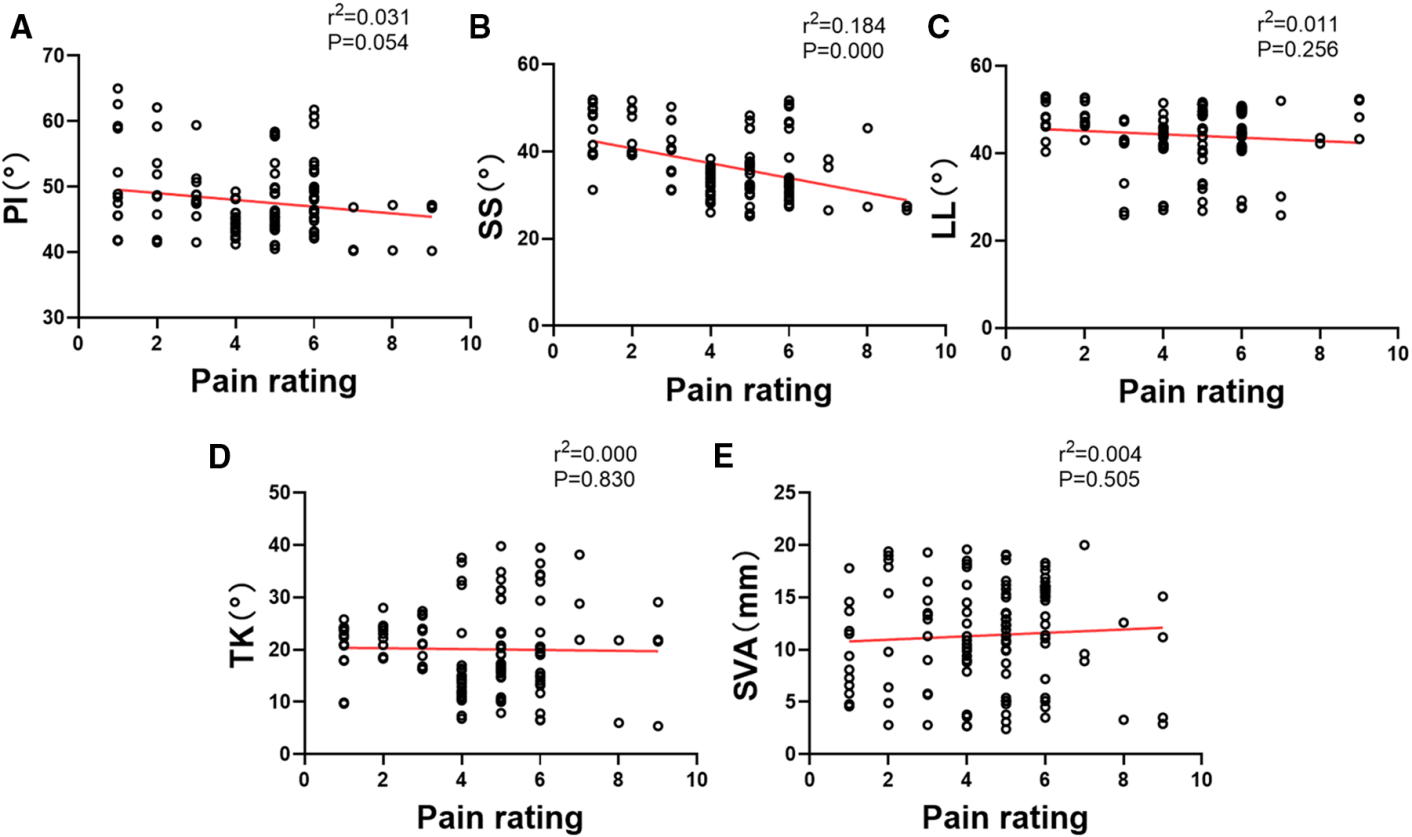
The correlation analysis of the pain rating and sagittal spino-pelvic alignment parameters.

The correlation analyses were carried out to assess the correlation between pain rating and each spino-pelvic alignment parameters above. In summary, there were no significant correlation between pain rating and PI (*r*^2 ^= 0.031, *P* = 0.054), LL (*r*^2 ^= 0.011, *P* = 0.256),TK (*r*^2 ^= 0.000, *P* = 0.830) and SVA (*r*^2 ^= 0.004, *P* = 0.505), while a significant negative correlation between pain rating and SS (*r*^2 ^= 0.184, *P* = 0.000). The linear regression of correlation analyses above were shown in Figure 3.

### The correlation between Roussouly spine classification and PD

**Figure 4:**
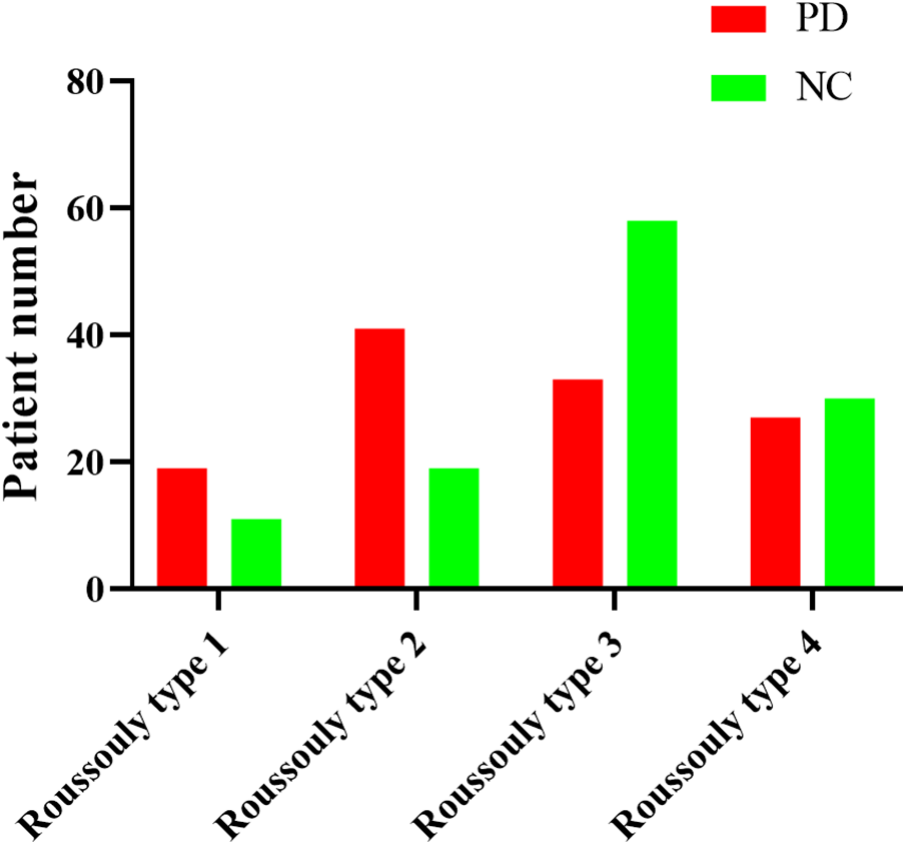
The patients number of PD group and normal group in each Roussouly spine classification type.

The PD patients and normal volunteers’ radiographic data were then classified according to their Roussouly spine classification. The Roussouly type 1 patient number of PD group and normal group were 19 and 11 respectively. As for Roussouly type 2, the patient number of PD group and normal group were 41 and 19. And the Roussouly type 3 patient number of PD group and normal group were 33 and 58, the Roussouly type 4 patient number of PD group and normal group were 27 and 30. The distribution of the patients number of each Roussouly type was shown in Figure 4.

**Figure 5:**
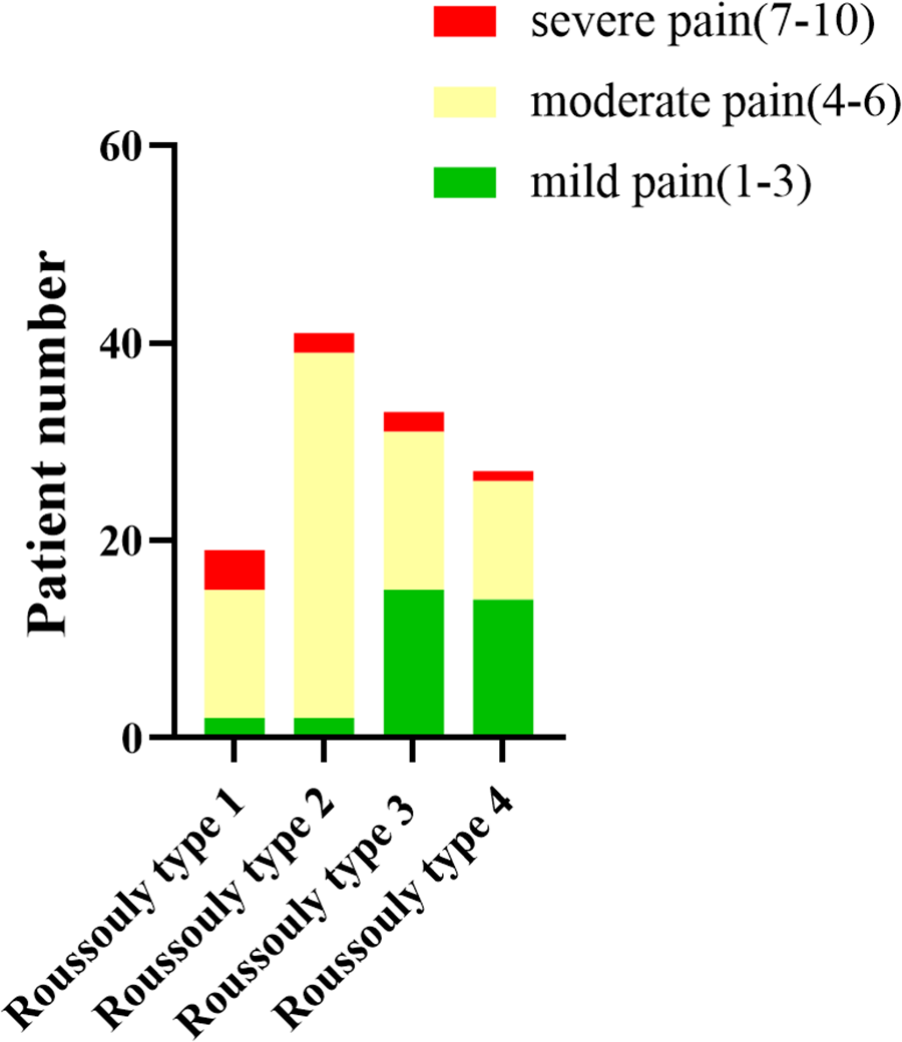
The PD patients with different pain rating classified with Roussouly type.

To further investigate the correlation between Roussouly classification of PD patients and pain rating, the different pain rating PD patients were sorted by Roussouly spine classification type. There were 2 mild pain patients, 13 moderate pain patients and 4 severe pain patients classified with Roussouly type 1. And 2 mild pain patients, 37 moderate pain patients and 2 severe pain patients were classified with Roussouly type 2. While 15 mild pain patients, 16 moderate pain patients and 2 severe pain patients were classified with Roussouly type 3. Besides, 14 mild pain patients, 12 moderate pain patients and 1 severe pain patients were classified with Roussouly type 4. The distribution of the PD patients with different pain rating classified with Roussouly type was shown in Figure 5.

## Discussion

In recent years, the sagittal spino-pelvic alignment or balance had received more and more attention from various scholars and spine surgeons. Several studies have investigated the importance of sagittal spino-pelvic alignment in spinal disorders such as lumbar spondylolisthesis (12–14), adolescent idiopathic scoliosis (15–18), and adult spinal deformity (19, 20). At the same time, the research on the etiology of primary dysmenorrhea mainly focuses on uterine over contraction (21), vasoconstriction (22, 23), inflammation and release of inflammatory mediators (24, 25), brain Imaging and stimulation of pain fibers (26–29).

The human species is characterized by bipedal walking, which has the advantage of freeing up upper limbs for other tasks, but at the expense of a more unstable overall balance. Indeed, other primates, unlike humans, move on all the four limbs. Their spine presents a single thoraco-lumbar curvature in kyphosis which provides more space for pelvic and abdominal organs and a better global sagittal balance (30). However, at present, there is no study that reported other primates or mammals have primary dysmenorrhea. Primary dysmenorrhea can be defined as a unique symptom to human females. So far, research on the etiology of primary dysmenorrhea has focused more on the pathophysiological factors. In this study, we innovatively discussed the relationship between primary dysmenorrhea and sagittal spino-pelvic alignment, which provided a new and original insights on the etiology of primary dysmenorrhea.

Our results firstly indicated that the PI, SS and LL in PD patients were significant lower than those in normal people (Table 2). In addition, we found that the PI and SS in patients with PD who experienced mild pain were significantly higher than those in patients with moderate pain (Table 3). Surprisingly, a significant negative correlation was found between PD patients' pain rating and SS (Figure 3), which indicated that the lower SS was, the higher pain rating would be. Besides, the distribution of Roussouly spine classification in normal and PD groups showed significant differences. There were more PD patients classified with Roussouly type 2, meanwhile the majority of normal people were classified with Roussouly type 3 (Figure 4). Moreover, a large part of PD patients with moderate pain were classified with Roussouly type 2 (Figure 5). The Roussouly type 2 was defined as that SS was less than 35°, the apex of the lumbar lordosis is located at base of the L4 vertebral body, and the both thoracic and lumbar curve were small, presenting a harmonious flat back appearance. Based on all the results above, it could conceivably be hypothesized that a lower SS and PI angle may be related to a worsen pain in PD patients.

**Figure 6:**
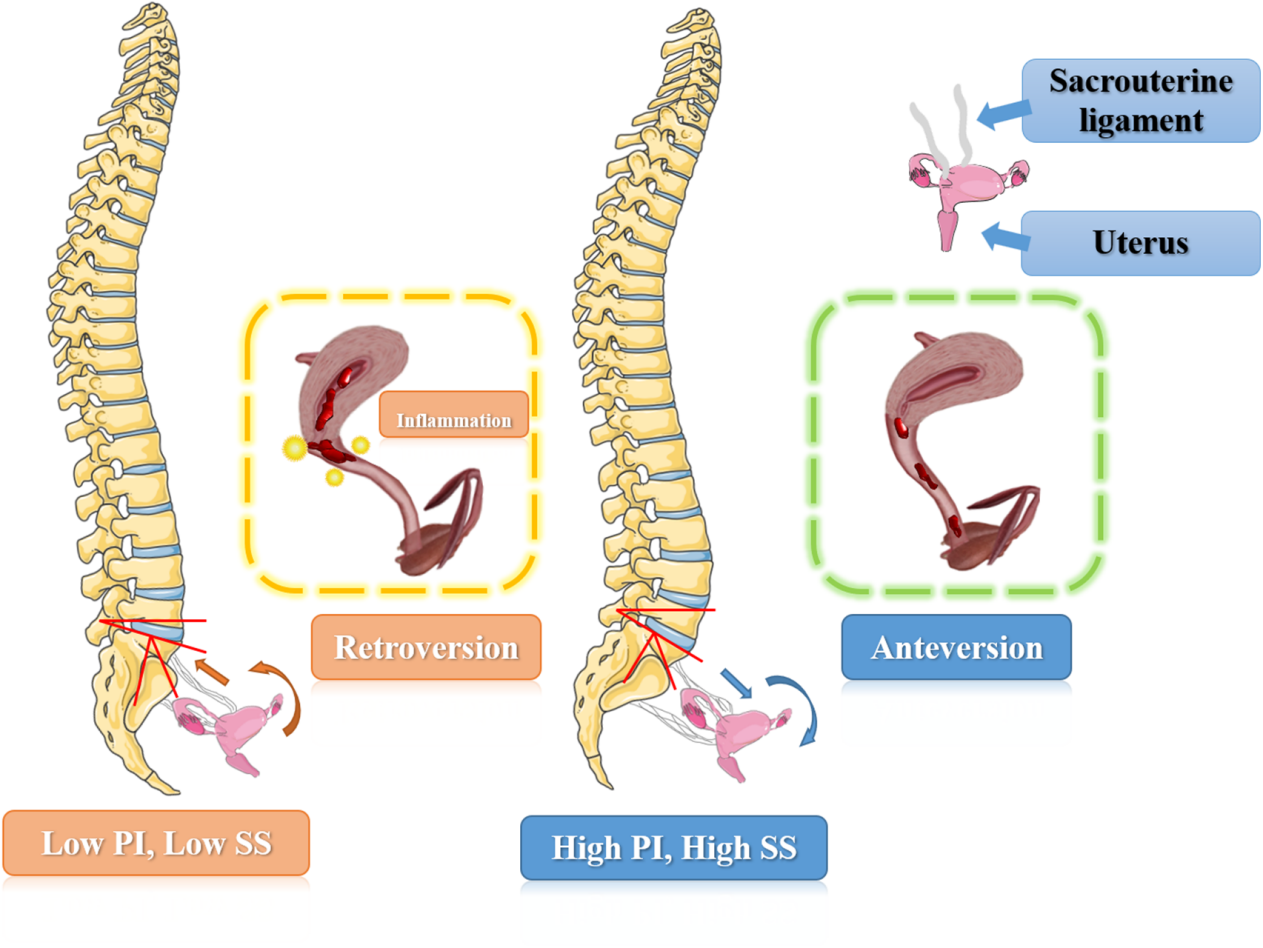
The schematic illustration of possible pathological mechanisms how spino-pelvic alignment parameters affect the primary dysmenorrhea symptoms.

According to the results we found and the hypothesis raised above, an implication of this is the possibility that a lower sacral slope and pelvic incidence may lead to a related smaller pelvic space and a retroversion of uterus. The excessive retroversion could lead to outflow obstruction of menstrual blood and uterine secretions, resulting in local inflammatory reaction, so as to stimulate uterine contraction and cause dysmenorrhea. On the other hand, a higher SS and PI could make uterus a relatively anteversion position, leading to a smooth outflow of menstrual discharge through vagina and therefore relieving the pain. The possible pathophysiological mechanisms were shown in Figure 6.

However, this study still has some limitations. The number of patient samples was limited in this retrospective study, a larger sample size was required to fully validate the hypothesis above. Furthermore, limited by the current gynecological ultrasound and abdominal CT examination require supine position, we were not able to assess the patients’ exact uterine position in a standing position, thus could not precisely verify the correlation between the spino-pelvic alignment parameters and pelvic organ position.

## Conclusion

In summary, we first reported the relationship between sagittal spino-pelvic alignment and primary dysmenorrhea. In PD patients, worsening pain may be correlated with lower SS and PI angles, which provided a fresh and unique perspective on the cause of primary dysmenorrhea.

## Data Availability

The raw data supporting the conclusions of this article will be made available by the authors, without undue reservation.
